# Challenges in Controlled Doping of NaMnF_3_:Yb^3**+**^, Er^3**+**^ Nanoparticles

**DOI:** 10.1021/acsomega.6c00594

**Published:** 2026-02-05

**Authors:** Oliver Bergmann, Simon Stiller, Nils Steuer, Moritz von zur Mühlen, Christina Graf

**Affiliations:** † 38968 Hochschule Darmstadt, Stephanstraße 7, Darmstadt 64295, Germany; ‡ EUt+ Institute of Nanomaterials and Nanotechnologies, EUTINN, European University of Technology, European Union, https://www.univtech.eu/eutinn; § Technical University Darmstadt, Peter-Grünberg-Str. 4, Darmstadt 64287, Germany

## Abstract

NaMnF_3_:Yb^3+^, Er^3+^ upconversion nanoparticles (UCNPs) are highly attractive for multimodal biomedical imaging because they combine a single red emission band with magnetic properties, allowing their use as *T*_1_ contrast agents in magnetic resonance imaging. However, compared to other magnetic or red-emitting UCNPs, their synthesis remains challenging, so they are rarely used despite their great potential. Syntheses based on hydrothermal approaches usually yield rather large or aggregated and often polydisperse and polymorphic particles. Reported thermal decomposition methods, in principle more suited to yield highly crystalline, small, and monodisperse UCNPs, are questionable because of the unclear phase and doping state of the NaMnF_3_ particles despite their strong red emission. This is due to the parallel formation of red luminescent NaYbF_4_ particles, which has not been sufficiently investigated. In this work, different thermal decomposition routes are studied, and the particles are analyzed to optimize them for high dopant integration and strong red luminescence at 655 nm. An optimized synthesis route for well-defined phase-pure particles in a size range from 20 to 25 nm was developed. This approach is based on separately preparing Mn^2+^, Er^3+^, and Yb^3+^ oleates and then introducing them into the reaction, followed by a purification step. This leads to phase-pure UCNPs and substantial increases in dopant concentrations as well as luminescence compared to reported synthesis routes. After their hydrophilic functionalization with polyacrylic acid, the ability of the particles to serve as magnetic resonance imaging (MRI) contrast agents with a high Mn^2+^ content was demonstrated by NMR relaxometry.

## Graphical abstract

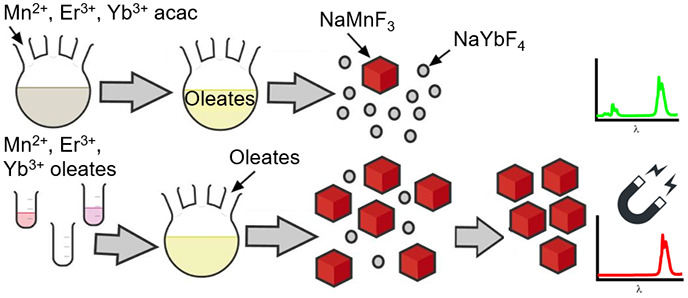

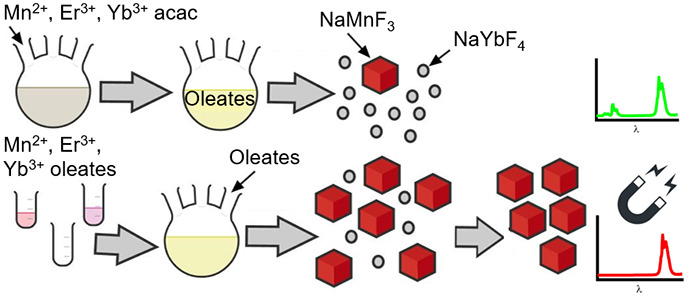

## Introduction

Due to their favorable characteristics, including low toxicity, deep tissue penetration, nonautofluorescence, and readily tunable emission, lanthanoid-doped upconversion nanoparticles (UCNPs) are attractive for a wide range of scientific fields.^1–11^ They have been employed in a multitude of applications, spanning from cancer treatment to temperature sensors, magnetic resonance imaging (MRI) contrast agents, and solar cells, among others.^12–18^

In recent years, there has been a notable shift toward tuning UCNP emissions to the red band or creating particle systems that exhibit a pure red-band emission under NIR excitation.^19–22^ This property is particularly advantageous in bioimaging applications, as the red emission can penetrate deeper into tissue than the usually dominant green emission of NaYF_4_:Yb^3+^, Er^3+^ UCNPs.^19,23^ For example, NaYF_4_:Yb^3+^, Er^3+^ particle systems with strong green emission at 545 nm and weak emission at 655 nm have been tuned to the desired red emission by doping with elements such as Mn^2+^ or Fe^3+^.^11,20,22,24^ Another approach is the development of UCNPs with a pure red emission.^19,25–27^ NaMnF_3_:Yb^3+^, Er^3+^ is a promising particle system that exhibits an intense red emission due to its high Mn^2+^ concentration and its capacity to enable nonradiative energy transfer from the ^2^H_9/2_ and ^4^S_3/2_ levels to the ^4^F_9/2_ level of the Er^3+^ ions via its ^4^T_1_ energy state.^25^ This results in a particle system with a strong red emission under 980/808 nm excitation.

Furthermore, NaMnF_3_:Yb^3+^, Er^3+^ particles can be employed as MRI *T*_1_ contrast agents and are, therefore, promising candidates for incorporation into multifunctional particle systems.^19,25,28^ Particles combining magnetic and upconversion properties can be used for multimodal imaging in vitro or in vivo, as well as parts of multifunctional drug delivery systems.^11,29–32^ Combining the upconversion properties of biocompatible lanthanoid ions with magnetic ions that provide *T*_1_ contrast in a single material, which could, moreover, later be used as part of a shell around a superparamagnetic core that serves as a *T*_2_ contrast agent, could therefore be highly beneficial. While most *T*_1_ contrast agents currently in use are based on gadolinium, which has seven unpaired electrons and is considered safe, concerns exist about their accumulation in the human body and the environment, as well as other side effects associated with Gd^3+^.^33–37^ Alternatives, such as manganese with its five unpaired electrons, have been studied and have gathered some attention as a possible substitute.^38–42^ NaMnF_3_:Yb^3+^, Er^3+^ nanoparticles, with their high manganese content, could, as such, prove to be an optimal system for a multifunctional particle strategy.

Previously, the synthesis of NaMnF_3_:Yb^3+^, Er^3+^ was predominantly based on the thermal decomposition of chloride or acetylacetonate salts.^19,25^ In these studies, the resulting dopant concentrations were not analyzed, and the complete phase composition remained unclear, making direct comparisons impossible. However, similar syntheses using this established route in our laboratory resulted in particles with low dopant incorporation and the formation of two distinct crystal phases, a smaller one consisting of undesired 5 nm particles of NaYbF_4_, as well as a larger phase of 25 nm diameter particles of the desired composition. Microwave-assisted syntheses of NaMnF_3_ particles of various sizes and morphologies have also been published in recent years.^43,44^ However, successful doping of these systems with lanthanoids was not reported. Additionally, room-temperature or hydrothermal approaches for NaMnF_3_ synthesis have been described, yielding relatively large particles (around 200 nm) that are mostly unsuitable for biological applications.^28,45–47^

This work presents an optimized high-temperature synthesis route for the production of highly doped NaMnF_3_:Yb^3+^, Er^3+^ nanoparticles. We optimized the doping level of the particles by varying the precursors used in the particle synthesis process. By switching from the established approach of using chloride or acetate/acetylacetonate salts in a one-pot synthesis to the use of separately preformed oleates, the increased incorporation of dopants by approximately 4-fold for Yb^3+^ and even up to 6-fold for Er^3+^ was reached. This results in significantly improved luminescence, particularly in the desired 655 nm red band of the spectrum. Furthermore, the effects of doping ratios on particle formation and luminescence intensity were examined. It was possible to identify the formation of a second particle phase, specifically at high dopant concentrations, and to develop a cleanup process that vigorously separates the particles, resulting in highly doped pure-phase particles.

## Results and Discussion

### Syntheses of NaMnF_3_:Yb^3+^, Er^3+^Nanoparticles from Acetylacetonates and Acetates

Initially, NaMnF_3_:Yb^3+^, Er^3+^ nanoparticles were prepared in a high-temperature decomposition synthesis according to the work of Ye et al.^25^ In a one-pot synthesis, Mn^2+^ acetylacetonate and the acetates of Yb^3+^ and Er^3+^ were first reacted with oleic acid to form the corresponding oleates and then converted without further purification with NH_4_F and NaOH. The total concentration of the two dopants Yb^3+^ and Er^3+^ was above 20%. These syntheses yielded predominantly particles with a rounded morphology and a diameter of 5 ± 1 nm (see TEM image in Figure S1 in the SI). The XRD pattern (Figure 1) shows two distinct phases, indicating that the obtained particles do not consist of the pure phase NaMnF_3_ (ICDD 00-018-1224) but are mainly NaYbF_4_ (ICDD 01-077-2043). Using the Scherrer formula, it was estimated that the NaMnF_3_ phase has a crystallite size of approximately 25 ± 2 nm, and the NaYbF_4_ phase has a crystallite size of approximately 5 ± 1 nm, as confirmed via TEM imaging (see below). Upon reduction of the dopant concentration below 20% Yb^3+^/2% Er^3+^, the percentage of NaMnF_3_ nanocubes increases compared to that of the NaYbF_4_ particles. These observations are consistent across all dopant concentrations, as can exemplarily be seen in the XRD in Figure 1. This finding is also supported by the corresponding TEM images in the Figures S1–S5 in the Supporting Information, which show a significant decrease in the number of cubes along with an increase in their size with an increasing dopant concentration in the reaction mixture, while the amount of small NaYbF_4_ particles increases significantly).

**Figure 1:**
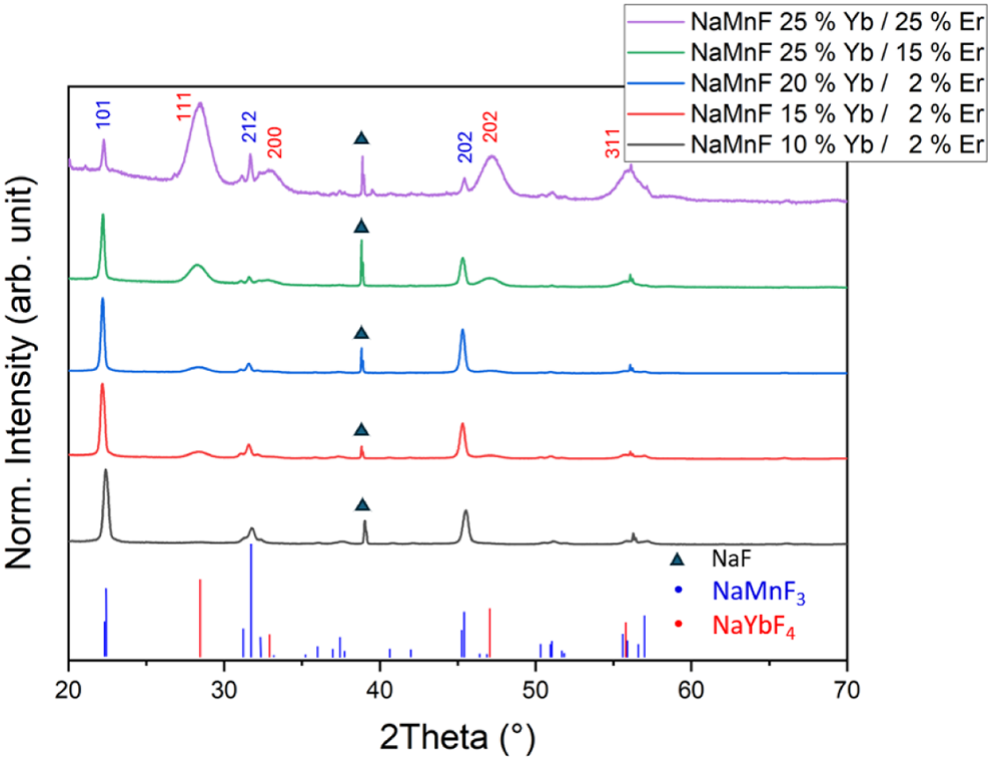
X-ray diffractograms of NaMnF_3_:Yb^3+^, Er^3+^ samples with a significant NaYbF_4_ phase synthesized via the acetate/acetylacetonate route at various dopant concentrations, showing an increase in NaYbF_4_ concentration as the dopant concentration in the reaction mixture increases. The ICDD patterns of pure NaMnF_3_ (ICDD 00-018-1224, blue pattern) and NaYbF_4_ (ICDD 01-077-2043, red pattern) are shown for comparison. The 200 reflexes of NaF are labeled with a black triangle. The measurements were carried out directly after the initial washing steps, without separating the NaF particles. TEM overview images of the synthesis are shown in the Supporting Information (Figures S1–S5). All diffractograms are normalized to the 101 reflex of NaMnF_3_.

These results show the formation of NaYbF_4_ particles as an unintended byproduct, a finding consistent with the observations made by Bai et al.,^28^ who also report the formation of small particles above specific dopant concentrations during the synthesis of Er^3+^/Yb^3+^-codoped NaMnF_3_ nanocubes. However, they did not further analyze the phases. Since doping at higher concentrations leads to the predominant formation of NaYbF_4_ particles, and NaMnF_3_ particles synthesized via this route have a low dopant concentration, alternative synthesis routes were explored.

### Improved Doping via the Use of Presynthesized Oleates

As Ye et al. reported in their work on the particle system, MnCl_2_ has a low reactivity toward the formation of Mn^2+^oleates.^25^ Therefore, Mn^2+^ acetylacetonate was used instead in their synthesis of NaMnF_3_:Yb^3+^, Er^3+^ nanoparticles.^25^ However, as our results with acetylacetonates and the predominant formation of NaYbF_4_ suggest, the reactivity of Mn^2+^ acetylacetonate toward oleic acid is also low compared to the highly reactive Yb^3+^ and Er^3+^ acetates, so the Yb^3+^ and Er^3+^ oleates are preferentially formed when the three metal precursors are converted together in a one-pot synthesis without any purification or separation step. Consequently, during the subsequent heat-up process, the byproduct NaYbF_4_ is largely formed instead of NaMnF_3_:Yb^3+^, Er^3+^ nanoparticles. To circumvent this problem, the three oleates of Mn^2+^, Yb^3+^, and Er^3+^ were synthesized separately and then employed in the heat-up synthesis as described above. The doping ratio was systematically investigated, i.e., the percentages of Yb^3+^ and Mn^2+^ in the reaction mixture were varied. In this way, it was possible to ensure that the immediate Mn^2+^, Yb^3+^, and Er^3+^ precursors were available in the desired stoichiometric ratio. This leads to an increase in Mn doping (see below) and a higher conversion rate. However, as the XRD data show, the formation of the byproduct NaYbF_4_ could not be completely suppressed (see Figure 2). The remaining NaYbF_4_ nanoparticles could then be completely removed via centrifugation in hexane after the synthesis due to their much smaller size of around 5 nm compared to the 5-times larger diameter of the NaMnF_3_:Yb^3+^, Er^3+^ NPs. The phase purity of the purified samples was confirmed by XRD. Figure 2 shows the X-ray diffractograms of a typical NaMnF_3_ sample prepared from separately prepared oleates directly after the synthesis and in its purified state, obtained by removing NaYbF_4_ via centrifugation. The resulting nanoparticles were compared with purified particles prepared directly from the corresponding metal acetylacetonates and acetates in a one-pot synthesis with the same dopant ratios. The elemental composition of the particles was analyzed via ICP-MS, and the results of this series of measurements are shown in Figure 3.

**Figure 2:**
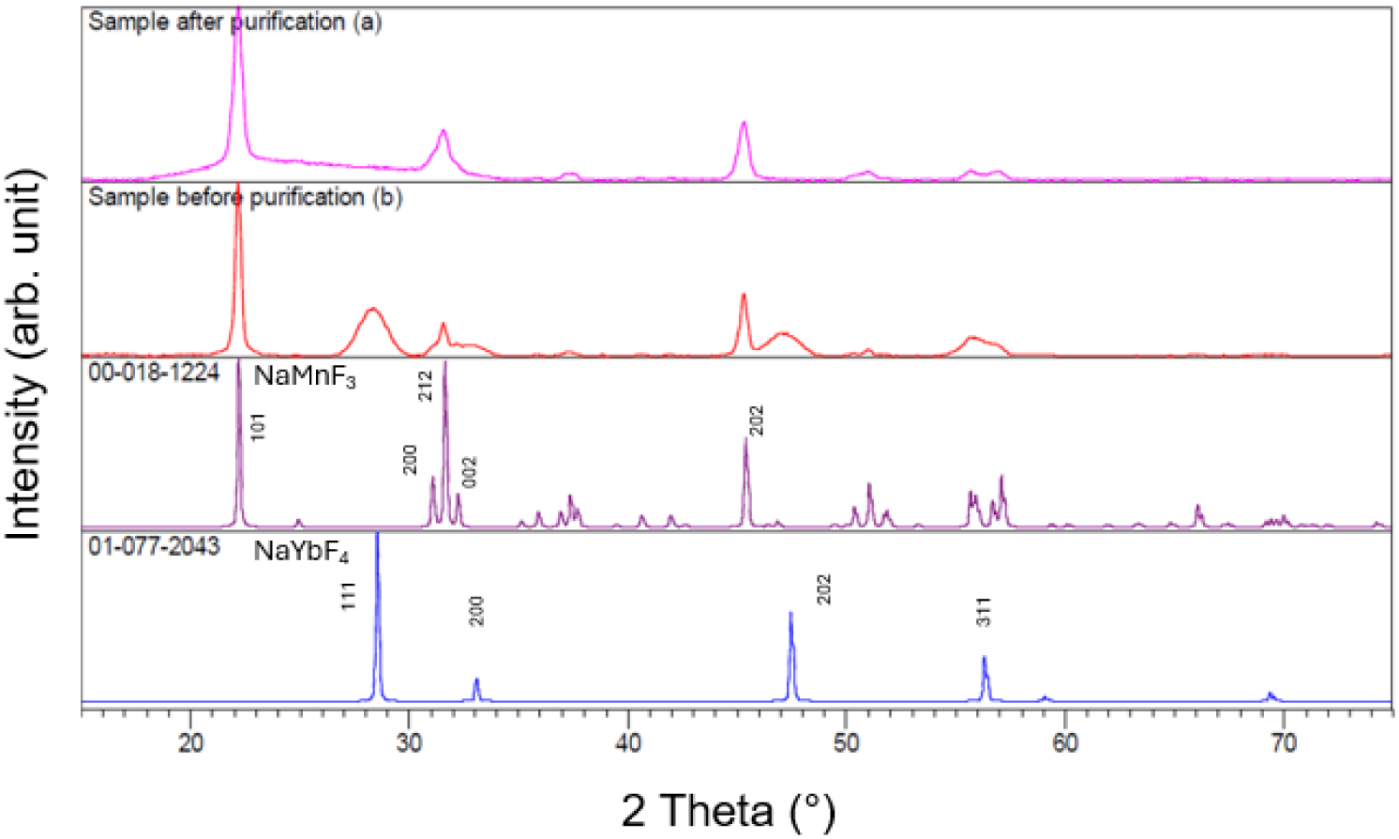
X-ray diffractograms of a NaMnF_3_:Yb^3+^, Er^3+^ sample with a NaYbF_4_ phase synthesized via the oleate route (25%/2% Yb^3+^/Er^3+^): (a) after separation of the NaYbF_4_ phase and (b) unpurified sample after precipitation and NaF removal, as well as the ICDD patterns for NaMnF_3_ (00-018-1224) and NaYbF_4_ (01-077-2043) corresponding to the shown diffractograms.

**Figure 3:**
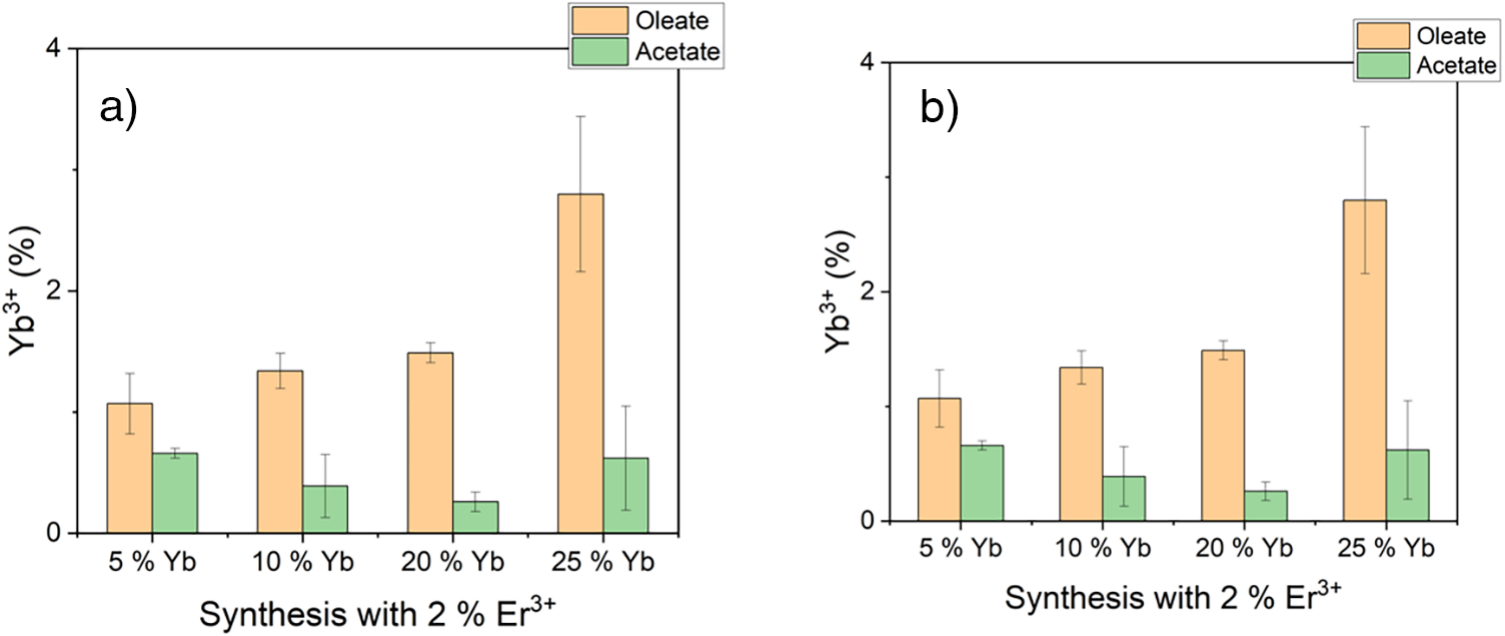
Results of the ICP-MS analysis of nanoparticles derived from syntheses conducted via the acetylacetonate and oleate routes: (a) measured concentration of Yb^3+^ and (b) measured concentration of Er^3+^ in the NaMnF_3_ nanoparticles as a function of the Yb^3+^ percentage in the synthesis. The erbium concentration in the reaction mixture was maintained at 2% throughout the series. The byproduct NaYbF_4_ was completely removed in all samples prior to the ICP-MS measurement.

As shown in Figure 2, the particles were successfully separated, and their diffraction patterns correspond well to the established standards. The distinctive, clearly separated reflexes associated with NaYbF_4_ (111, 200, and 202) are absent in the diffraction pattern of the purified sample. The results in Figure 3 demonstrate that the use of the presynthesized oleates increases the doping level by up to 4-fold for Yb^3+^ and up to 6-fold for Er^3+^ when compared to syntheses using the acetylacetonate route with the same dopant ratio in the reaction mixture. This outcome can be explained by the relatively limited quantity of Mn^2+^ oleate, which is formed in the one-pot synthesis in the presence of the more reactive Yb^3+^ acetate (see Figure 1), which predisposes the formation of NaYbF_4_ as a byproduct, thereby reducing the generation of doped NaMnF_3_ nanoparticles. In contrast, the stringent purification and washing steps applied to the oleates after their separate synthesis result in more defined formation of the desired oleate phases and amounts. Although the synthesis with acetates/acetylacetonates was theoretically sound, it suffered from poor dopant incorporation into the particles. Transmission electron microscopy (TEM) confirms the successful purification and separation of the two phases, as shown in Figure 4.

**Figure 4:**
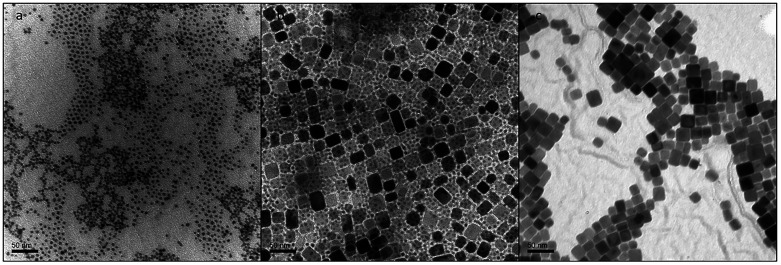
TEM images of a typical synthesis: (a) separated NaYbF_4_ phase, (b) NaMnF_3_:Yb^3+^, Er^3+^and NaYbF_4_ nanoparticles after precipitation without separation by size, and (c) purified NaMnF_3_:Yb^3+^, Er^3+^ phase.

Transmission electron microscopy (TEM) confirms the successful purification and separation of the two phases, as shown in Figure 4.

Even though the use of presynthesized oleates ensures that Mn^2+^, Yb^3+^, and Er^3+^ precursors are available in the desired ratio, a considerable proportion of the Yb oleate is converted to NaYbF_4_, and a significant fraction of the NH_4_F is converted to NaF. This may be explained by the relatively high reaction enthalpy of the formation of NaMnF_3_. Although no exact thermodynamic data are available for this reaction, according to the literature, NaMnF_3_ has a rather distorted orthorhombic perovskite structure compared to other perovskites.^48^ Consequently, the lattice energy for its formation is −18.86 eV/mol. In contrast, e.g., it is −25.89 eV for the more stable KMnF_3_.^49^ Liu et al. observed in a microwave synthesis of undoped NaMnF_3_ microparticles that at low nucleation temperatures of 260 or 270 °C, predominantly NaF is obtained instead of NaMnF_3_.^44^ A similar observation was made by Du et al. in a thermolysis-based study of undoped NaMnF_3_, where, below 280 °C, NaF is also the main product.^50^ In contrast, the standard formation enthalpy (−1503.3 kJ/mol) of NaYbF_4_ ^51^ is even more negative than the corresponding value for NaF (−546.20 kJ/mol),^52^ indicating the high thermodynamic stability of this compound. Accordingly, Chen et al. observed in a hot-injection synthesis of NaYbF_4_ nanoparticles that the cubic α-phase of NaYbF_4_ already forms at 180 °C.^53^ Therefore, it is feasible that the formation of these undesired byproducts cannot be suppressed during the synthesis of NaMnF_3_:Yb^3+^, Er^3+^ nanoparticles. Alternatively, a hot-injection approach was carried out to try to prevent the formation of these unwanted byproducts at lower temperatures during the heat-up process. However, at high temperatures (≥300 °C) required for the formation of NaMnF_3_ nanoparticles, the cubic α-NaYbF_4_ phase quickly transforms into its hexagonal β-phase.^53,54^ Since β-NaYbF_4_ nanoparticles are much larger than α-NaYbF_4_ nanoparticles and are in the same size range as the cubic NaMnF_3_ particles, it is impossible to separate both particle types.

Figure 5 shows the upconversion emission spectra of the NaMnF_3_:Yb^3+^, Er^3+^ phase, as well as the NaYbF_4_:Er^3+^, Mn^2+^ phase after their separation. The NaMnF_3_:Yb^3+^, Er^3+^ particles show an intense red emission around 655 nm, corresponding to the ^4^F_9/2_ → ^4^I_15/2_ transition of Er^3+^, with the green emission, corresponding to the ^4^S_3/2_ → ^4^I_15/2_ transition, being absent. In contrast, the NaYbF_4_ particles show a distinct green emission. The process of tuning the UC emission toward the red ^4^F_9/2_ → ^4^I_15/2_ is well-studied and desirable for bioimaging applications.^25,27,55,56^ The absence of the green emission in the NaMnF_3_ samples results from the high Mn^2+^ concentration which favors nonemissive transitions from the Er³^+^^4^S_3_/_2_ to the Mn²^+^^4^T_1_ level and, from there, to the ^4^F_9/2_ state.^55^ Obviously, the concentration of Mn^2+^ in the secondary synthesized NaYbF_4_ particles is insufficient to achieve complete suppression of the green emission (see Table S1 in the SI).

**Figure 5:**
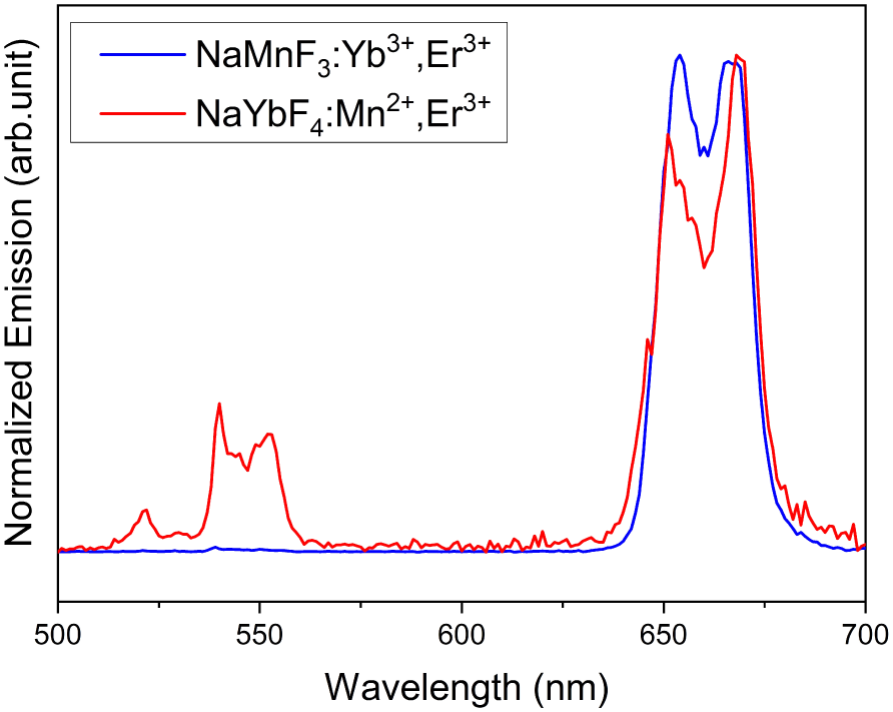
Typical room temperature UC emission spectra of NaMn(96.5 ± 1%)­F_3_:Yb^3+^ (2.5 ± 0.6%), Er^3+^ (1 ± 0.4%), as well as NaYb(74 ± 3%)­F_4_:Mn^2+^ (17 ± 1%), Er^3+^ (8.5 ± 0.5%) particles in *n*-hexane, excited at 980 nm (power density: 6.6 ± 0.5 W/cm^2^). The emission at 655 nm was normalized to 1 for comparison.

Furthermore, the luminescence decay at 655 nm was investigated (see Figure S7 in the Supporting Information), and it was observed that the NaYF_4_:Yb^3+^, Er^3+^ particles exhibit a decay profile with prolonged decay times of τ = 206 ± 8 μs. The luminescence decay of NaYbF_4_ is relatively fast, with a time constant of τ = 19 ± 2 μs. The observed discrepancy between the two crystal phases can be attributed to their size and the resulting distance between the excited ions and the crystal surface, as well as the associated reduction in phonon modes.^57^

### Optimization of Doping Level

The results above (see Figure 3) demonstrate that the incorporation of up to 2.8 ± 0.6% Yb^3+^ into the particles is feasible when the Yb^3+^ concentration in the reaction mixture is increased by up to 25%, while maintaining a fixed Er^3+^ concentration of 2%. Using higher amounts of Yb^3+^ in the reaction mixture decreases the yield of NaMnF_3_ particles by up to 80%, compared to syntheses with lower doping concentrations. This is because high Yb^3+^ concentrations favor the formation of NaYbF_4_. To further improve the synthesis, the effects of elevated erbium concentrations on particle formation and luminescence properties were investigated. In upconversion systems such as NaYF_4_:Yb^3+^, Er^3+^ nanoparticles, dopant concentrations of approximately 18%/2% Yb^3+^/Er^3+^ are commonly used and have been well-researched.^58,59^ In contrast, higher Er^3+^ concentrations often led to quenching effects. Figure 3b proves that in the present NaMnF_3_ system, only a small fraction of the experimentally used erbium amount is incorporated into the particles. Therefore, a series of syntheses with varying ratios of Er^3+^ and Yb^3+^ has been conducted to determine whether the incorporation of the dopants, as well as IR luminescence, can be enhanced by increasing the amount of Er^3+^. The extent of erbium and ytterbium incorporation into NaMnF_3_ nanoparticles prepared with a fixed amount of Yb^3+^ in the reaction mixture was quantified by ICP-MS to test the efficacy of incorporating increasing amounts of Er^3+^ ions into the nanocubes. As presented in Figure 6, the incorporation of Yb^3+^ into the nanocrystals varies considerably despite the concentration being fixed at 25% in all reaction mixtures.

**Figure 6:**
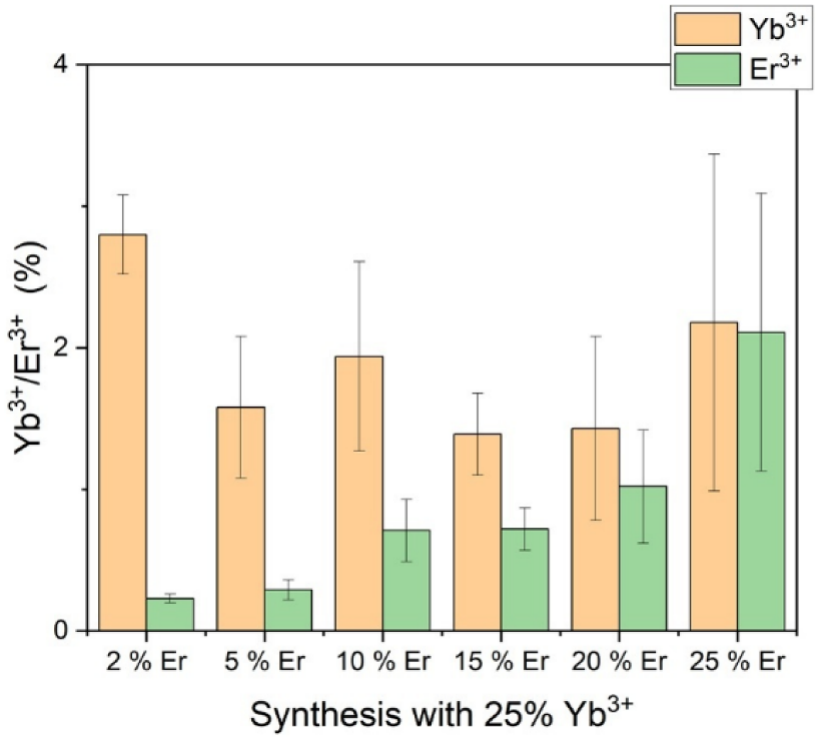
ICP-MS data for a series of NaMnF_3_ nanoparticles with a fixed Yb^3+^ content of 25% and varying Er^3+^ amounts in the reaction mixture. The measured concentrations of Yb^3+^ and Er^3+^ in the nanoparticles are plotted as a function of the Er^3+^ percentage in the synthesis.

As Figure 6 indicates, the incorporation of Er^3+^ correlates positively with increasing Er^3+^ availability. However, as the concentration of Er^3+^ increases, the concentration of Yb^3+^ decreases. It is hypothesized that this is due to the chemical similarity of the two ions, particularly their similar crystal ion radii of Yb^3+^ (86.8 pm) and Er^3+^ (89.0 pm), which results in comparable rates of integration into the NaMnF_3_ crystal. While this allows for a wide variety of doped particles and higher doping concentrations of erbium and ytterbium, it also results in reduced total emission of the particles if the erbium concentration becomes too high, as shown in Figure 7. However, it should be noted that the doping levels achievable with this approach, as shown in Figure 5, are significantly higher than those of the classical acetylacetonate route.

**Figure 7:**
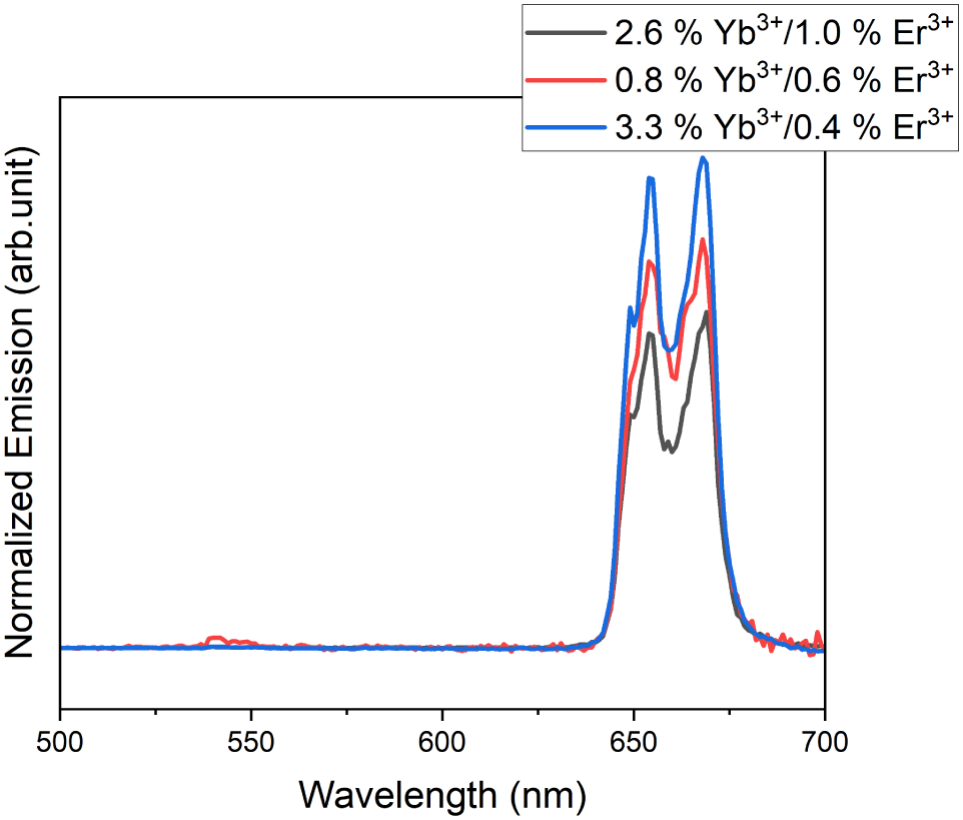
Room temperature UC emission spectra of NaMnF_3_:Yb^3+^, Er^3+^ nanoparticles in *n*-hexane, excited at 980 nm (power density: 6.6 ± 0.5 W/cm^2^) at different doping concentrations. The spectra are normalized to the absorption at 976 nm. The concentration of the dopants (Yb^3+^, Er^3+^) in the reaction mixtures was top to bottom (25%/20% (black), 25%/10% (red), 25%/2% (blue)).

An increase in the doping ratio, in conjunction with a reduction in the overall emission, also results in the enhanced formation of the NaYbF_4_ phase at high doping concentrations, as shown in Figure 8. A comparison of the diffractograms of samples from syntheses with 2%, 5%, and 10% Er and a constant Yb content of 25% shows an increase in the (111) reflex of NaYbF_4_. It is therefore recommended that doping be carried out at lower concentrations, around 25%/2% Yb^3+^/Er^3+^, as our findings suggest that a high Yb^3+^ ratio, together with a low Er^3+^ ratio, is favorable for high luminescence intensity.

**Figure 8:**
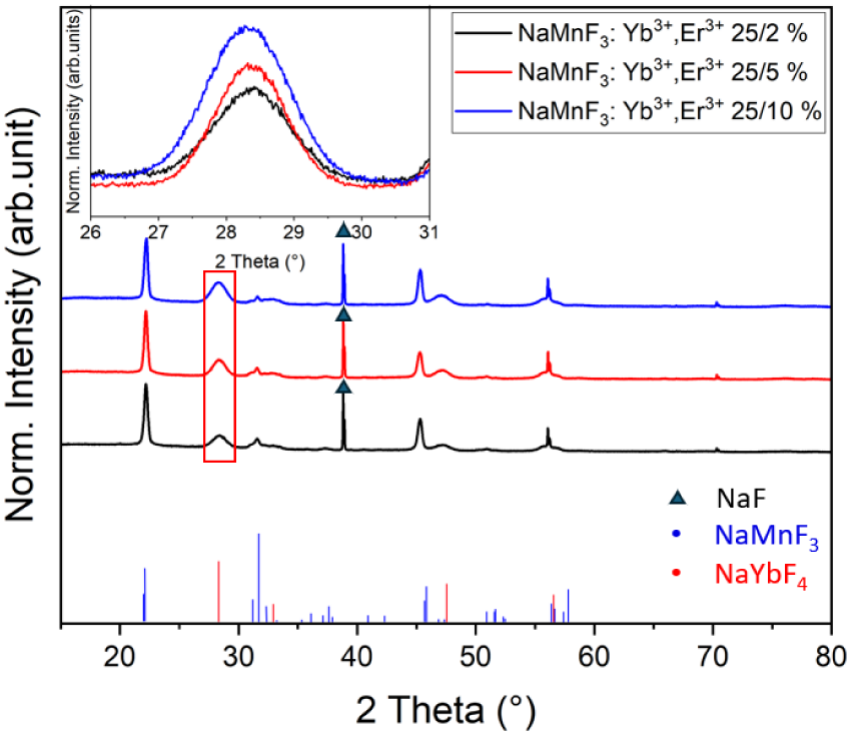
XRD data of NaMnF_3_:Yb^3+^, Er^3+^ nanoparticles with three different doping ratios from oleate-based synthesis without prior purification of the phases, as well as the corresponding ICDD patterns for NaMnF_3_ (00-018-1224) and NaYbF_4_ (01-077-2043). The 200 reflexes of NaF (01-088-2299) are labeled with a black triangle. All diffractograms are normalized to the 101 reflex of NaMnF_4_. The inset shows an enlarged view of the 111 reflex of NaYbF_4_, as indicated by the red frame in the main image.

### NMR Relaxometry Measurements

In order to ascertain the viability of the NaMnF_3_:Yb^3+^, Er^3+^ particles as a *T*_1_ contrast agent, their longitudinal relaxation times were measured via NMR relaxometry after the particles were functionalized with PAA in DEG according to a synthesis described by Liu et al.^60^ to stabilize them in polar solvents. Afterward, the particles were stored in water containing 0.1 M NaF before measurement. An *r*_1_ value of 2.06 ± 0.01 mM^–1^ s^–1^ was calculated from a linear fit of 1/*T*_1_ versus the Mn^2+^ concentration (Figure 9). This shows that the particles can, in principle, be used as an MRI *T*_1_ contrast agent.

**Figure 9:**
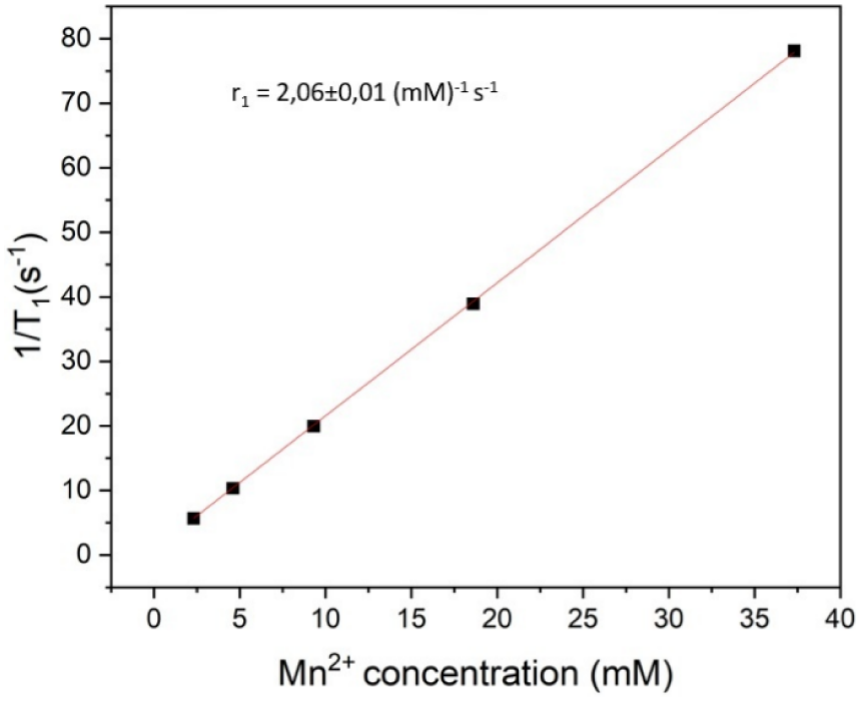
Relaxivity plot of 1/*T*_1_ vs Mn concentration of PAA functionalized NaMnF_3_:Yb^3+^, Er^3+^ (Mn^2+^: 91.44%, Yb^3+^: 7.95%, Er^3+^: 0.608%) particles in aqueous dispersion.

## Conclusions

A synthesis route has been developed that allows the incorporation of the dopants Yb^3+^ and Er^3+^ into phase-pure NaMnF_3_ particle systems in a heat-up synthesis. This synthesis method is classical, a one-pot synthesis that is constrained by the low reactivity of Mn^2+^, resulting in an increased formation of the byproduct NaYbF_4_. We demonstrate that the doping of the system with Yb^3+^ can be increased by up to four times and with Er^3+^ by up to six times by separately preparing the oleates of Mn^2+^, Er^3+^, and Yb^3+^ and then introducing them into the heat-up synthesis. This procedure is followed by a simple purification step. The resulting phase-pure particles exhibit pure red luminescence at approximately 655 nm upon excitation with a 980 nm laser. Furthermore, it has been shown that a dopant ratio of 25% Yb^3+^ and 2% Er^3+^ in the reaction mixture yields particles with enhanced luminescence compared to samples prepared with higher Er^3+^ concentrations. Furthermore, significant, doping at this lower ratio compared to increased Er^3+^ amounts greatly reduces byproduct formation, favoring an almost pure product that is easily separated during cleanup. Due to their high Mn^2+^ content of 37–39%, the particles can be used as *T*_1_ contrast agents in MRI. Their suitability for this imaging technique was demonstrated by NMR relaxivity measurements after the particles had been made dispersible in water by functionalization with polyacrylic acid.

## Experimental Details

### Chemicals

NH_4_F (>99%), erbium­(III) acetate (99.9%), erbium­(III) chloride (99.9%), ytterbium­(III) acetate (99.9%), ytterbium­(III) chloride (99.9%), methanol (99.8%), oleic acid (90%), manganese acetylacetonate, diethylene glycol (99%), and polyacrylic acid (PAA) (average *M*_v_ = 1800 g/mol) were purchased from Sigma-Aldrich. Acetone, cyclohexane, hexane, and ethanol were purchased from Merck. Manganese­(II) chloride (>98%) was purchased from TCI. Sodium hydroxide (>99%) was received from Carl Roth. All chemicals and solvents were used without further purification.

### Synthesis of Manganese Oleate and Lanthanoid Oleates

The synthesis of manganese oleate was adapted from Park et al.^63^ 7.9 g of MnCl_2_ were added to 22.6 g of previously synthesized Na oleate (see Supporting Information for details). 40 mL EtOH, 30 mL ultrapure water, as well as 70 mL hexane were added to the mixture. The resulting solution was heated to 60 °C for 4 h under vigorous stirring. A separatory funnel was used to separate the resulting phases, and the oil phase was washed three times with ultrapure water. At this step, it is essential to take care to prevent oxidation of the Mn oleate; otherwise, the pink product will turn deep red. Therefore, purification and storage were carried out in an argon atmosphere. The resulting viscous phase was transferred into a 250 mL round-bottom flask and first dried at a rotary evaporator (55 °C, 150 mbar) to remove acetone and hexane and then thoroughly dried on a Schlenk line for 6 h at 0.003 mbar. The resulting product was stored under argon at −18 °C.

The synthesis of the lanthanoid oleates was adapted from Na et al.^64^ 1.94 g (5 mmol) of YbCl_3_ or 1.91 g (5 mmol) of ErCl_3_ and 4.6 g (15 mmol) of Na oleate were dissolved in 35 mL EtOH, 10 mL ultrapure water, and 35 mL hexane. All subsequent steps are identical to those previously described in the synthesis of Mn oleate.

### Synthesis of NaMnF_3_: Yb^3+^,Synthesis of NaMnF: Yb, Er^3+^ Nanocubes Using Acetylacetonates and Acetates

For the synthesis of NaMnF_3_:Yb^3+^, Er^3+^ nanocubes using manganese and lanthanoid acetylacetonates based on Ye et al.,^25^ 0.78 mmol of manganese acetylacetonate, 0.2 mmol of ytterbium acetate, and 0.02 mmol erbium acetate were added into a 50 mL three-necked round-bottom flask.^25^ 7 mL oleic acid and 15 mL 1-octadecene were added, and the reaction mixture was flushed with argon. The solution was then heated to 50 °C and degassed to remove any water residues. Afterward, the reaction mixture was heated to 160 °C under argon and stirred vigorously for 1 h. During this time, the solution changed from colorless to dark yellow. The solution was then cooled to 50 °C, and 4 mmol of NH_4_F and 2.5 mmol of NaOH dissolved in 5 mL methanol were added to the cooled solution. The solution was stirred for another 30 min to initiate nucleation. The methanol was removed in vacuum (10^–4^ mbar, 50 °C) before the solution was heated to 300 °C under an argon atmosphere for 1 h. During the heating, the solution turned brown. After cooling, the particles were precipitated with ethanol (4:1), centrifuged (3000 g, 15 min), and redispersed in 1–2 mL *n*-hexane. This step was repeated twice, and finally, the particles were stored in hexane under an argon atmosphere.

### Synthesis of NaMnF_3_ Nanocubes Using Manganese and Lanthanoid Oleates

The synthesis is largely similar to that previously reported by Ye et al.,^25^ except that the oleates were prepared separately prior to the synthesis, and an additional purification step was added. First, the metal oleates in the required molar ratios (for example, 0.78 mmol of Mn oleate, 0.2 mmol of Yb oleate, and 0.02 mmol of Er oleate (78/20/2%)) were added to 7 mL of oleic acid and 15 mL of 1-octadecene and heated to 80 °C at a rate of 10 K/min until all metal oleates were dissolved. The previously colorless solution turns bright red during this step. It took about 5 min for the oleates completely dissolve, then the reaction mixture was cooled to 50 °C, and 4 mmol of NH_4_F and 2.5 mmol of NaOH dissolved in 5 mL of methanol were added, and the reaction mixture was stirred for 30 min (450 rpm). The methanol was again removed by degassing (50 °C) before heating the reaction mixture to 300 °C for 1 h. When the reaction temperature reached 200 °C, the reaction mixture changed from a turbid brown-orange to a bright yellow. Subsequently, the nanoparticles were precipitated by adding ethanol (30 mL), centrifuged (5000g, 10 min), and then redispersed in 6 mL hexane and washed twice with ethanol (30 mL). Finally, the nanocubes were stored in hexane (6 mL) under an argon atmosphere.

For purification of the two phases, the particles were dispersed in hexane in a centrifugation tube (10 cm height) and centrifuged at 6300 g for 4 h to remove the 4–5 nm-sized NaYbF_4_ particles. The resulting pellet was redispersed in hexane. This process was repeated three times; the particles were then checked for purity via XRD and TEM and stored in *n*-hexane under argon.

### Hydrophilic Functionalization with PAA (Polyacrylic Acid)

The functionalization was carried out largely as previously reported by Liu et al.^60^ First, 0.5 g of PAA was mixed with 10 mL of DEG and heated to 110 °C under an Ar atmosphere. After a clear solution was formed, 15 mg particles dispersed in 2 mL of hexane were injected into the solution. The reaction mixture was then kept at 110 °C for 30 min under an argon atmosphere. Afterward, the dispersion was heated to 240 °C for 2 h. After cooling, the dispersion was mixed with EtOH in a 1:2 ratio and centrifuged at 8500 g for 4 h to ensure complete sedimentation of the functionalized UCNPs. The particles were redispersed in a 1:1 mixture of ultrapure water and EtOH (5 mL) using an ultrasonic bath (Bandelin SONOREX SUPER RK 512 H, 35 kHz). Subsequently, they were centrifuged and redispersed two more times (8500 g, 3 h). The particles were then stored in ethanol and later transferred into ultrapure water for further analysis.

### Characterization

The size and morphology of the samples were characterized by TEM (Zeiss EM 109, acceleration voltage: 80 kV). The samples were prepared by dispersing 0.5 g/L nanoparticles in hexane. A droplet of the dispersion was dried on a carbon-coated copper grid (Plano GmbH, 400 mesh). The UC luminescence spectra were recorded using a spectrofluorometer FS5 from Edinburgh Instruments with a slit that provided a spectral resolution of 1 nm without diluting the samples. The colloidal UCNPs were excited at 980 nm by using a continuous-wave laser diode with tunable laser power in the range of 0–2 W. All measurements were carried out at a power density of 6.6 ± 0.5 W/cm^2^. UC decay curves were measured with the same spectrofluorometer, with the laser set to pulse until 2000 counts were collected at 655 nm. Quartz glass cuvettes (QS Suprasil, 5 mm, Hellma) were used in all measurements. NIR absorption spectra were measured undiluted in a standard fluorescence quartz glass cuvette (QS Suprasil, 5 mm, Hellma) between 1100 and 930 nm with an Agilent Cary 5000 UV–vis NIR spectrometer. The X-ray powder diffraction patterns of the UCNPs were recorded using a Bruker D8 1000 W with an X-ray wavelength of 1.54 Å. The angle range of the measurements was 10 to 80°. Samples were prepared on a glass slide (LABSOLUTE microscope slides). The crystallite size was calculated from the broadening of a peak in the diffraction patterns using the Scherrer equation, under consideration of the instrumental broadening. The latter is small compared to the full width at half-maximum of the reflexes. ICP-MS (PlasmaQuant MS Elite) was used to determine the elemental composition of the UCNPs. Sample preparation was performed by dissolving the nanoparticles (4 mg) in aqua regia (1 mL) and subsequent dilution to 20 mL with H_2_O. Relaxometry was measured with a Bruker Avance III HD (9.4 T) in water using a variant of the classical inversion–recovery method with a weak gradient during the recovery delay to suppress radiation-damping contributions.^61,62^ All measurements were carried out at room temperature.

## Supplementary Material



## Notes

A detailed description of the sodium oleate synthesis, an additional TEM image of UCNPs from the acetate/acetylacetonate synthesis, X-ray diffractograms, and luminescence decay profiles (PDF)
