# Supplementary material for: Challenges in Controlled Doping of NaMnF3:Yb3+ , Er3+ Nanoparticles
Source: ACS Omega. 2026 Feb 5;11(6):10868–77. doi: 10.1021/acsomega.6c00594 (PMC12917643; doi:10.1021/acsomega.6c00594)
Supplement: Supplementary file 1 [file ao6c00594_si_001.pdf]

Supporting Information

Challenges in Controlled Doping of $NaMnF_3$: Yb^{3+}, Er^{3+} - Nanoparticles

*Oliver Bergmann,[a,b,c] Simon Stiller,[a,b] Nils Steuer,[a,b] Moritz von zur Mühlen,[a,b] and Christina Graf[a,b]**

a. Hochschule Darmstadt, Stephanstraße 7, 64295 Darmstadt, Germany

b. EUt+ Institute of Nanomaterials and Nanotechnologies EUTINN, European University of Technology, European Union

c. Technical University Darmstadt, Peter-Grünberg-Str. 4, 64287 Darmstadt

Synthesis of Sodium Oleate:

The synthesis of sodium oleate was carried out based on Graf et al.[1] First, 6 g of NaOH and 45 g of oleic acid were added to 75 mL of ultrapure water in a 250 mL flask. Then, the reaction mixture was stirred (80 rpm) at 50°C for 2 h. The resulting suspension was then transferred into a beaker containing 100 mL EtOH and dissolved using an ultrasonic bath (Bandelin SONOREX SUPER RK 512 H). After this, the reaction mixture was poured into another beaker containing 500 mL of cold (8°C) acetone to precipitate the sodium oleate. The solid Na oleate was then transferred to a

suction filter and washed three times with cold (8°C) acetone, collected, and dried under vacuum. It was stored under argon at -18°C for further use.

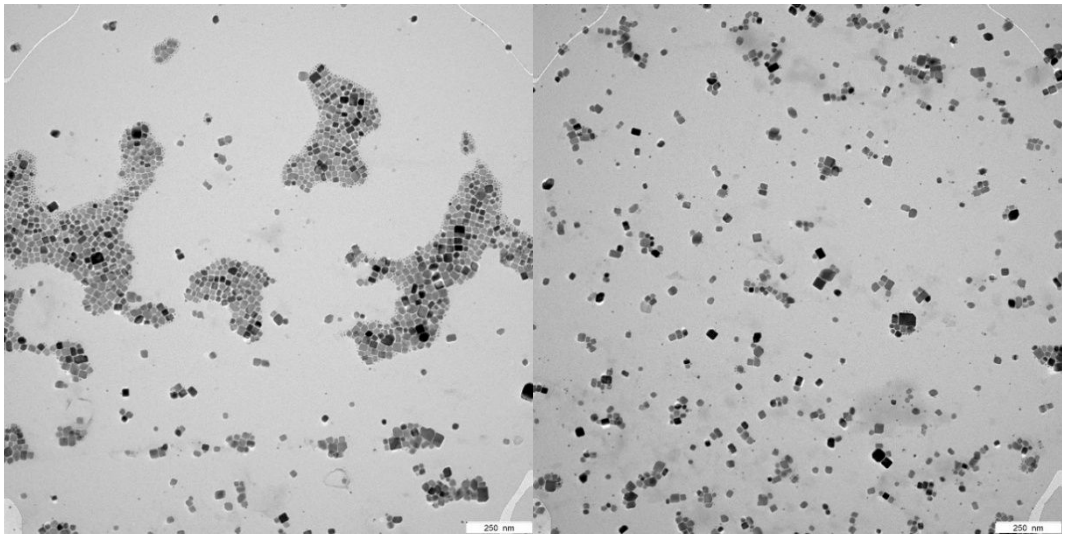

Figure S1. TEM images of a sample prepared by the acetylacetonate route, showing $NaYbF_4$ particles with a diameter of about 5±1 nm as well as cubic $NaMnF_3$ particles. This is a typical result for this synthesis at high doping ratios around 10/2 % Yb^{3+}/Er^{3+}. (The corresponding diffractogram is shown in Fig. 1).

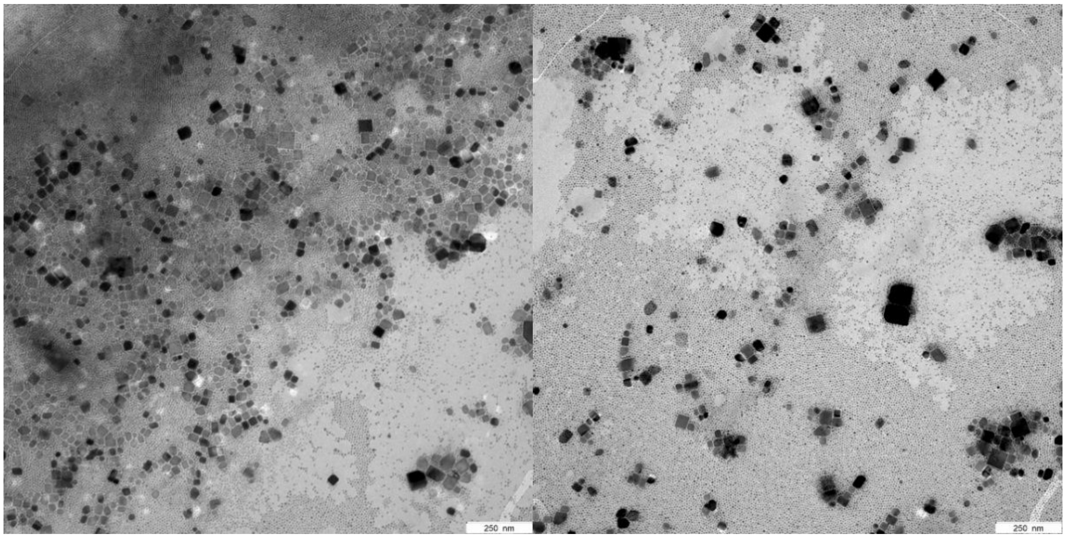

Figure S2. TEM image of a sample prepared by the acetylacetonate route, showing $NaYbF_4$ particles with a diameter of about 5±1 nm as well as cubic $NaMnF_3$ particles. This is a typical

result for this synthesis at high doping ratios around 15/2 % Yb^{3+}/Er^{3+}. (The corresponding diffractogram is shown in Fig. 1).

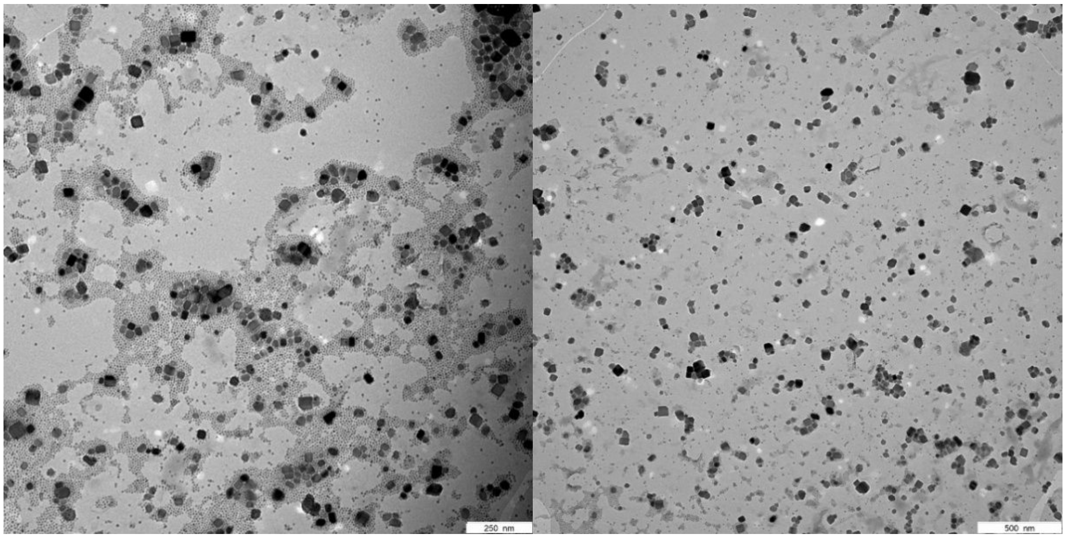

Figure S3. TEM image of a sample prepared by the acetylacetonate route, showing $NaYbF_4$ particles with a diameter of about 5±1 nm as well as cubic $NaMnF_3$ particles. This is a typical result for this synthesis at high doping ratios around 20/2 % Yb^{3+}/Er^{3+}. (The corresponding diffractogram is shown in Fig. 1).

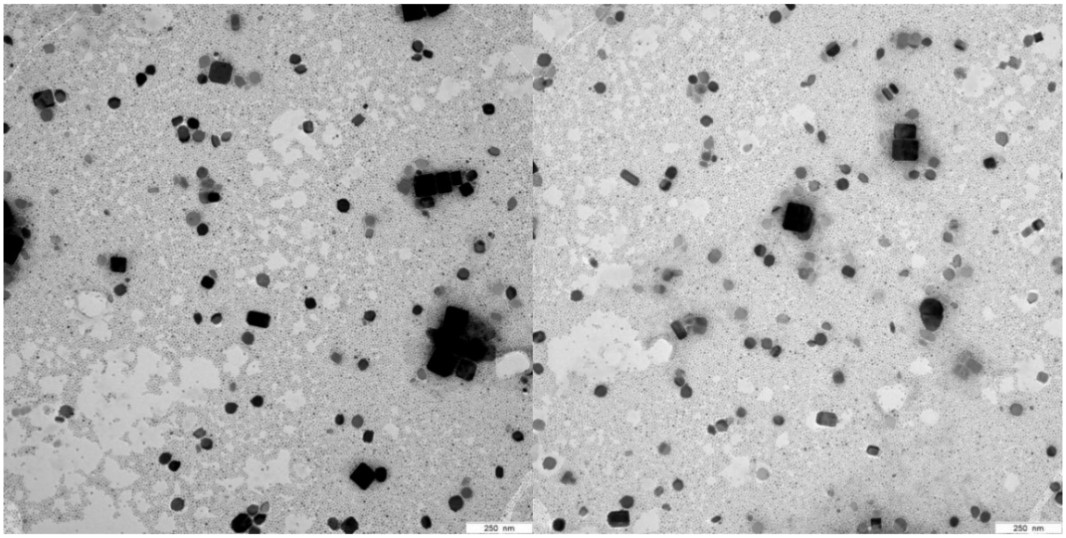

Figure S4. TEM image of a sample prepared by the acetylacetonate route, showing $NaYbF_4$ particles with a diameter of about 5±1 nm as well as cubic $NaMnF_3$ particles. This is a typical

result for this synthesis at high doping ratios around 25/15 % Yb^{3+}/Er^{3+}. (The corresponding diffractogram is shown in Fig. 1).

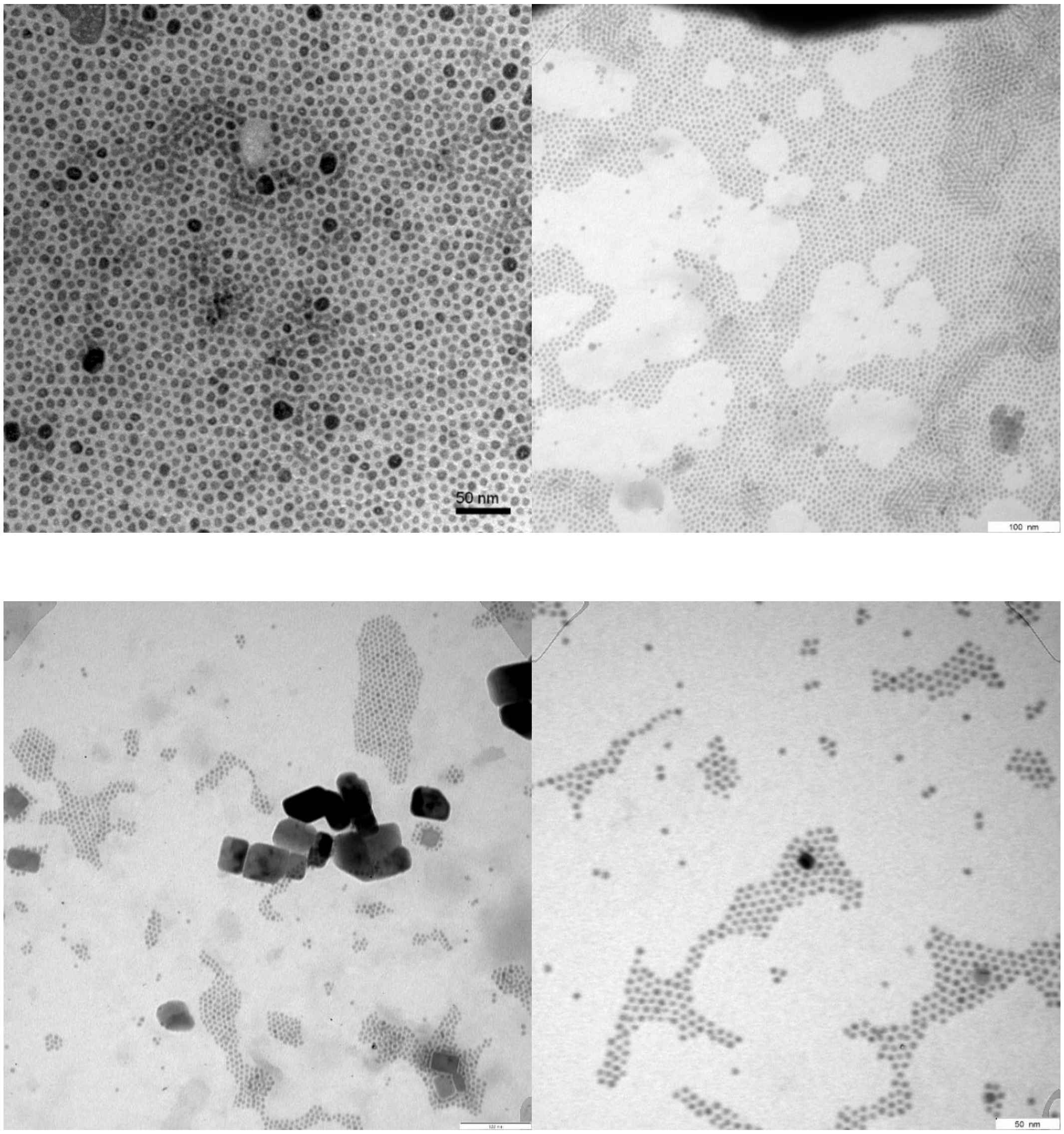

Figure S5. TEM images of a sample prepared by the acetate/acetylacetonate route, showing mainly $NaYbF_4$ particles with a diameter of about 5±1 nm. This is a typical result for this synthesis at high doping ratios around 25/25 % Yb^{3+}/Er^{3+}. (The corresponding diffractogram is shown in Fig. 1).

Table S1. ICP-MS results of two different $NaYbF_4$ samples after purification in mol%

**Sample**	**Mn %**	**Yb %**	**Er %**
NaYbF	17.2±0.8	74.3±3.7	8.5±0.4

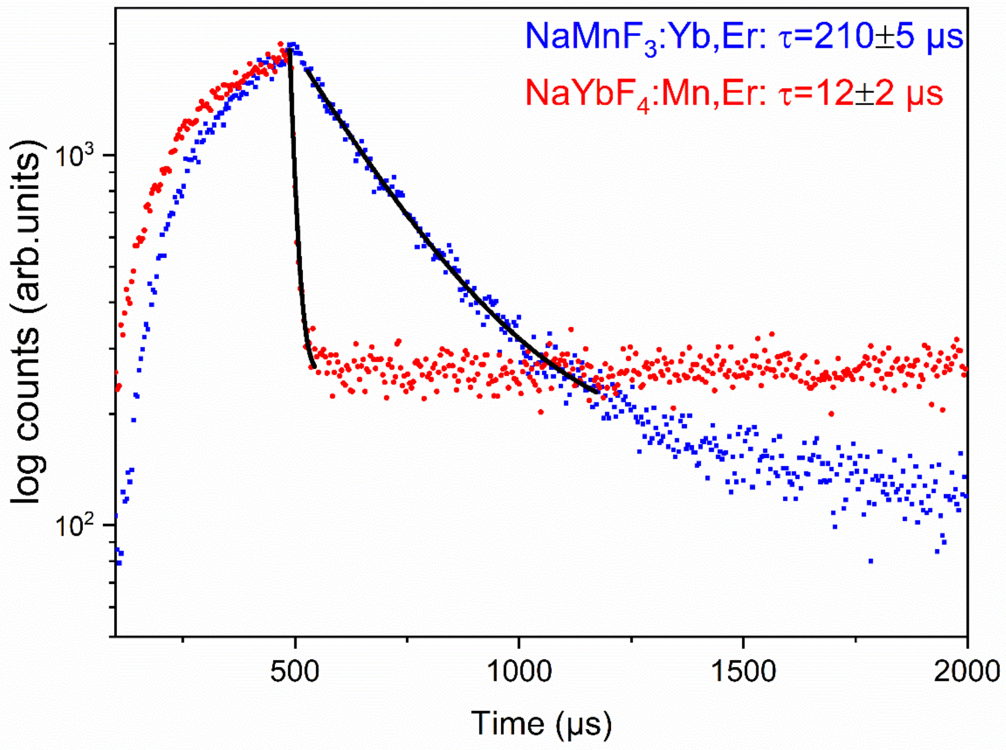

Figure S7. Luminescence decay profiles of $NaMnF_3$: Yb^{3+}, Er^{3+} and $NaYbF_4$: Yb^{3+}, Er^{3+} nanoparticles in n-hexane recorded at λ= 980 nm and a power density of 6.6±0.5 W/cm^2. The fits are single-exponential decays.

References

1 C. Graf, C. Goroncy, P. Stumpf, E. Weschke, C. Boeglin, H. Ronneburg and E. Rühl, Local Magnetic and Electronic Structure of the Surface Region of Postsynthesis Oxidized Iron

Oxide Nanoparticles for Magnetic Resonance Imaging, J. Mater. Chem. C, 2015, 119, 19404–19414.
